# Combination of EP4 antagonist and checkpoint inhibitors promotes anti-tumor effector T cells in preclinical tumor models

**DOI:** 10.1186/2051-1426-3-S2-P350

**Published:** 2015-11-04

**Authors:** Xingfeng Bao, Diana Albu, Kuan-Chun Huang, Jiayi Wu, Natalie Twine, Kenichi Nomoto, Mary Woodall-Jappe

**Affiliations:** 1 Eisai Inc., Andover, MA, USA

## 

Immunotherapies targeting the immune checkpoint receptors have shown great promise for a subset of cancer patients. However, robust and safe combination therapies are still needed to increase the benefit of cancer immunotherapy and bring it to broader patient populations. We have recently shown that E7046, a specific EP_4_ antagonist, possesses significant anti-tumor growth activity in multiple preclinical tumor models through modulating myeloid cells including tumor associated macrophages (TAMs) and myeloid-derived suppressor cells (MDSCs) (AACR 2015, poster #275). Here we evaluated the anti-tumor activities of E7046 in combination with the checkpoint inhibitors anti-CTLA4 and anti-PD1 antibodies, and also with E7777, a recombinant IL-2/diphtheria toxin fusion protein, in immunogenic CT26 and poorly immunogenic 4T1 tumor models. In the CT26 model, concomitant treatment of E7046 and anti-PD1 led to pronounced tumor growth inhibition, with 40% of the mice rendered stably tumor free, while either single agent produced mostly tumor growth inhibitory activity and only an occasional tumor-free animal. In the same model, markedly improved anti-tumor activity was observed for the combination of E7046 and E7777, with up to 20% of animals rendered stably tumor free, compared with only modest anti-tumor activity of either single agent treatment alone. In the 4T1 model, combining E7046 and anti-CTLA4 resulted in a nearly complete tumor growth inhibition; either agent alone had only modest growth inhibitory activity. In the tumor microenvironment of both models, an effective anti-tumor immune response was induced by the combination treatments including E7046 as indicated by the robust accumulation and activation of CD8 cytotoxic T cells (CTL) and/or significantly improved ratio of activated GZMB^+^CD8^+^ CTL vs CD4^+^CD25^+^Foxp3^+^ Treg cells. In addition, combination treatments including E7046 in tumor-bearing mice showed no additional gross toxicity compared with immune checkpoint inhibitors alone or E7777 alone. Taken together, these results demonstrated a superior activity of combinational therapies including E7046 over immune checkpoint inhibitors or E7777 alone with acceptable toxicity in preclinical models, and therefore candidates for combination trials in patients. IND filing number of E7046 is 125272.

